# Biological Evaluation, Molecular Docking, MD Simulation Study and Synthesis of (Z)‐N’‐(3‐phenoxybenzylidene)‐2‐(substituted‐amino aryl) Acetohydrazid

**DOI:** 10.1002/jbt.71114

**Published:** 2026-09-23

**Authors:** Navin B. Patel, Deepti S. Ghaisas, Vatsal M. Patel, Faiyazalam M. Shaiikh, Mohit Agrawal, Mohd Imran, Gildardo Rivera, Hetal I. Soni

**Affiliations:** ^1^ Department of Chemistry, Organic Research Laboratory Veer Narmad South Gujarat University Surat India; ^2^ Department of Chemistry C. B. Patel Computer College & J. N. M. Patel Science College, Affiliated to Veer Narmad South Gujarat University Surat India; ^3^ School of Medical & Allied Sciences, K.R. Mangalam University Gurugram India; ^4^ Center for Health Research Northern Border University Arar Saudi Arabia; ^5^ Laboratorio de Biotecnología Farmacéutica Centro de Biotecnología Genómica, Instituto Politécnico Nacional Reynosa Mexico

**Keywords:** antimicrobial activity, anti‐protozoa, anti‐tubercular H37RV, biphenyl ether, molecular docking study, Schiff's base

## Abstract

In order to investigate their possible biological activity, a number of new Schiff base compounds based on diaryl ether, specifically 2‐(substituted‐arylamino)‐N′‐(3‐phenoxybenzylidene) acetohydrazides, were created. IR, ^1^H NMR, and ^13^C NMR spectrum studies were used to characterize all substances. A structure–activity link influenced by the kind of substituents on the aryl moiety was suggested by the evaluation of the synthesized derivatives’ in vitro biological activity, which showed significant activity patterns among substituted analogs. Molecular docking studies demonstrated favorable binding interactions of the most active compounds with the selected biological target, supported by key hydrogen bonding and hydrophobic interactions within the active site. Furthermore, molecular dynamics simulations confirmed the stability of the ligand–protein complexes, indicating their potential as promising lead molecules. The novelty of this study lies in the design of hybrid diaryl ether–Schiff base derivatives and their combined experimental and computational evaluation, providing insights into their biological potential and guiding future optimization.

## Graphical abstract

Synthesis and biological evaluation (antibacterial, antifungal, anti‐tuberculosis, antiprotozoal) and *in silico molecular docking* of biphenyl ether based Schiff's base analogues.

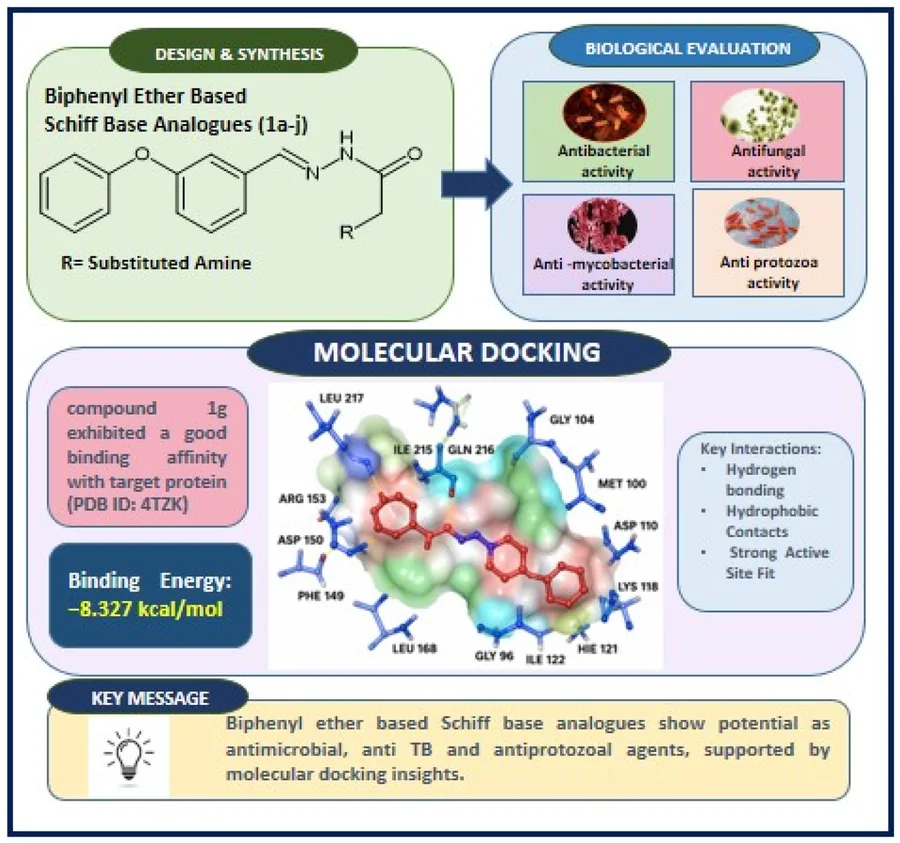

## 1 Introduction

In the search for new drugs, natural products and their structural motifs have long been a valuable source of physiologically active molecules [1, 2, 3]. The development of new therapeutic compounds with enhanced pharmacological qualities is still motivated by their structural variety [4]. Because of their wide range of biological functions, heterocyclic systems with nitrogen, oxygen, and sulfur atoms are very important. Natural compounds like Riccardin and Vancomycin contain diaryl ether scaffolds, which are a significant class of bioactive motifs [5, 6]. These substances are extensively studied in medicinal chemistry and display a variety of pharmacological characteristics. Another significant family that is well‐known for its diverse biological activities and potent metal‐binding properties are schiff bases, which are created when primary amines condense with carbonyl compounds [7, 8, 9, 10, 11, 12, 13, 14, 15].

According to reports, they contain antibacterial, anti‐inflammatory, antioxidant, and anticancer qualities [14, 15, 16, 17, 18, 19, 20, 21]. A possible method for creating new bioactive chemicals with increased activity is the combination of Schiff base moieties and diaryl ether. Computational methods like molecular docking and molecular dynamics (MD) simulations have emerged as useful tools for comprehending ligand‐protein interactions and forecasting complex stability in recent years.

The current study used molecular docking and MD simulations to design and synthesize a number of Schiff base derivatives based on diaryl ether, which were then evaluated in silico against a chosen biological target. This effort aims to investigate their binding interactions, evaluate complex stability, and shed light on their possible biological activities.

## 2 Result and Discussion

### 2.1 Chemistry

**Scheme 1:**
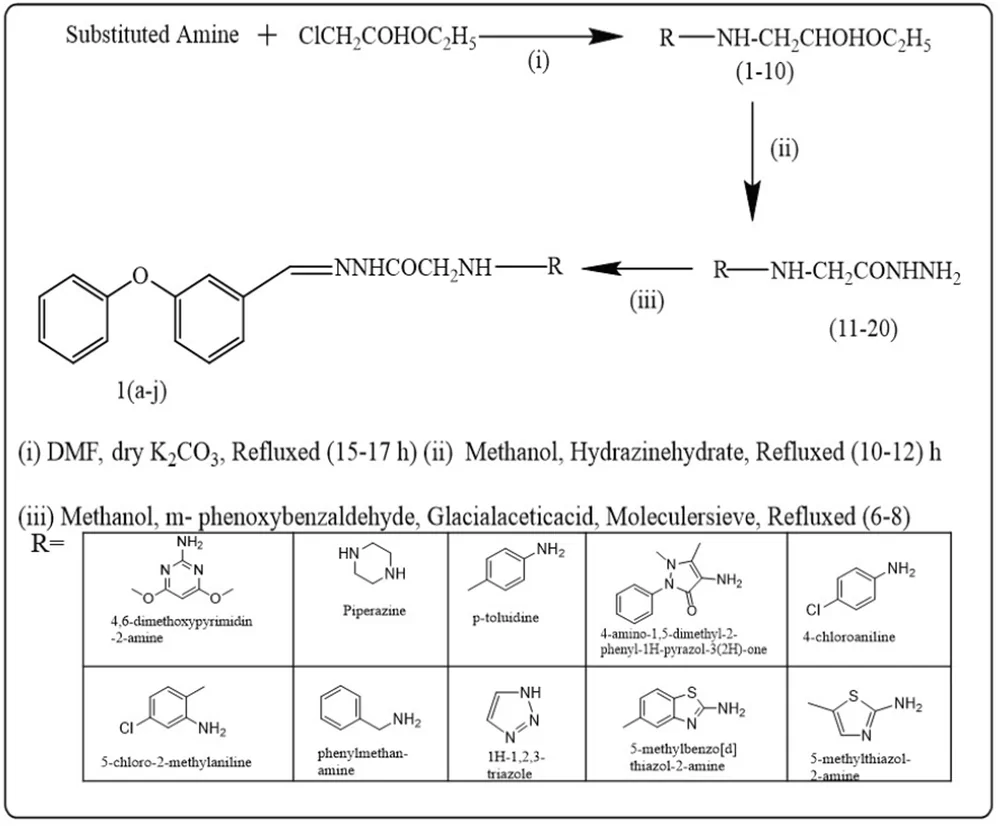
Synthesis of (Z)‐2‐(substituted‐arylamino)‐N’‐(3‐phenoxybenzylidene) aceto‐hydrazide 1(a‐j).

Scheme 1 The synthetic route to the target compounds 1a–j was accomplished through a three‐step procedure. Initially, ethyl 2‐(substituted arylamino)acetates (A) were synthesized via SN^2^ nucleophilic substitution of ethyl chloroacetate with the corresponding substituted aromatic amines. These intermediates were subsequently converted into 2‐(substituted arylamino)acetohydrazides (B) upon treatment with hydrazine hydrate. Final condensation of the hydrazide intermediates with m‐phenoxybenzaldehyde afforded the desired Schiff base derivatives, namely 2‐(substituted arylamino)‐N′‐(3‐phenoxybenzylidene)acetohydrazides (1a–j).

The structures of all synthesized compounds were confirmed by IR, ^1^H NMR, ^13^C NMR, and mass spectrometry.

In the IR spectra of intermediates (A), a characteristic N–H stretching band of the secondary amine was observed around ~3390 cm^−1^. The presence of the ester functionality was confirmed by a strong absorption band at ~1740 cm^−1^ corresponding to C = O stretching, along with bands at ~1220 cm^−1^ and ~1030 cm^−1^ attributed to C–O and C–O–C stretching vibrations, respectively. Upon conversion to the corresponding acetohydrazides (B), the disappearance of the ester carbonyl band and the appearance of a new amide carbonyl absorption in the region ~1650–1680 cm^−1^ confirmed successful hydrazide formation. Additionally, broad bands in the region ~3200–3300 cm^−1^ were observed due to N–H stretching vibrations of the hydrazide moiety.

The formation of the final Schiff base derivatives (1a–j) was evidenced by the appearance of a characteristic azomethine (C = N) stretching band in the region ~1580–1620 cm^−1^ in the IR spectra, along with the retention of the amide carbonyl band around ~1660 cm^−1^. Aromatic ether (C–O–C) stretching vibrations were observed near ~1240 cm^−1^, confirming the presence of the phenoxy moiety.

The ^1^H NMR spectra of the synthesized compounds showed a distinct singlet corresponding to the azomethine proton (–CH = N–) in the downfield region at δ ~8.5–8.7 ppm, confirming Schiff base formation. The amide proton (–CONH–) appeared as a singlet around δ ~8.0–8.5 ppm, while additional NH protons were observed as broad singlets in the region δ ~8.5–11.0 ppm due to hydrogen bonding. Aromatic protons resonated as multiplets in the range δ ~6.8–7.8 ppm. The methylene protons of the –CO–CH_2_– group appeared as a singlet around δ ~3.8–4.1 ppm. Substituent‐specific signals such as methyl and methoxy groups were observed at their expected chemical shift regions.

The ^13^C NMR spectra further supported the proposed structures. The amide carbonyl carbon resonated in the region δ ~165–170 ppm, while the azomethine carbon (C = N) appeared around δ ~140–150 ppm. Aromatic carbons were observed between δ ~115–140 ppm, and the ether‐linked aromatic carbon (C–O–C) appeared downfield at δ ~ 155–160 ppm. The methylene carbon (–CO–CH_2_–) was observed around δ ~50–60 ppm, along with signals corresponding to substituent carbons at their respective positions.

Mass spectral analysis of the synthesized compounds exhibited molecular ion peaks corresponding to their respective molecular weights, along with [M + H]^+^ peaks, confirming their molecular formulas. The observed mass data were in good agreement with the calculated values for all derivatives.

Overall, the combined spectral data conclusively confirmed the successful synthesis of the targeted 2‐(substituted arylamino)‐N′‐(3‐phenoxybenzylidene) acetohydrazide derivatives.

### 2.2 Pharmacology

#### 2.2.1 In Vitro Antimicrobial Activity

The broth micro dilution method was used to determine the minimum inhibitory concentration (MIC) of the synthesized compounds. MIC is defined as the lowest concentration that causes ≥ 99% suppression of microbial growth. The majority of compounds shown moderate action against both Gram‐positive (*S. aureus*, *S. pyogenes*) and Gram‐negative (*P. aeruginosa*, *E. coli*) bacteria. When compared to the usual medication Ampicillin, a number of compounds had good antibacterial action, including 1 d (*P. aeruginosa*), 1e (*E. coli*), 1 f (*S. aureus*), 1 g, 1i, and 1j (*P. aeruginosa*) (MIC = 100 µg/mL) against specific strains. With the exception of compound 1 g against *Candida albicans* (MIC = 100 µg/mL) when compared to the conventional medication Nystatin, the majority of other derivatives showed moderate efficacy whereas antifungal activity was generally modest. The antibacterial results are summarized in (Table 1).

**TABLE 1:** In vitro antimicrobial activity (MIC, µg/mL) of the compounds 1(a–j).

Code	Antibacterial activity (MIC)				Antifungal activity (MIC)		
	Gram negative		Gram positive				
	*E. coli*	*P. aeruginosa*	*S. aureus*	*S. pynogenus*	*C. albicans*	*A. niger*	*A. clavatvs*
	MTCC 442	MTCC 441	MTCC 96	MTCC 443	MTCC 227	MTCC 282	MTCC 1323
	**(µg/mL)**				**(µg/mL)**		
1a	200	250	250	250	1000	1000	1000
1b	150	250	200	250	250	500	500
1c	200	500	250	250	1000	1000	> 1000
1 d	250	100	125	500	500	1000	1000
1e	100	200	250	500	500	1000	> 1000
1 f	250	250	100	500	250	500	500
1 g	100	200	125	250	100	1000	1000
1 h	200	125	200	500	200	500	500
1i	250	100	200	100	1000	1000	> 1000
1j	250	100	125	200	1000	500	500
**Drug**	**µg/mL**						
Ampicillin	100	100	250	100	—	—	—
Chloramphenicol	50	50	50	50	—	—	—
Ciprofloxacin	25	25	50	50	—	—	—
Norfloxacin	10	10	10	10	—	—	—
Nystatin	—	—	—	—	100	100	100
Greseofulvin	—	—	—	—	500	100	100

#### 2.2.2 In Vitro Anti‐Protozoa Activity

Table 2 summarizes the anti‐protozoal activity against Trypanosoma cruzi and Leishmania mexicana. Activity was categorized as weak (> 20 µg/mL), moderate (10–20 µg/mL), and good (IC⋅_0_ < 10 µg/mL). Compound 1e exhibited moderate activity against T. cruzi (IC^2^_0_ = 15.32 µg/mL) and good activity against L. mexicana (IC⋅_0_ = 6.70 µg/mL). While the other compounds were either weak or inert against L. mexicana, compound 1c showed moderate activity (IC^2^_0_ = 13.52 µg/mL). The potency of standard medications (miltefosine and nifurtimox) was higher (IC^2^_0_ = 1–7 µg/mL).

**TABLE 2:** Primary inhibition (IC_50_ µg/mL) against *M. tuberculosis H37Rv*, *L. mexicana* and *T. cruzi* of compound 1(a–j).

Sample Id	Anti‐mycobacterial activity	Antiprotozoal activity (µg/mL)	
	% Inh at conc.50 (µg/mL)		
	*H37Rv*	*L. mexicana* MNYC/BZ/62/M379	*T. cruzi* MHOM/MX/94/Ninoa
1a	24	> 50	> 50
1b	24	> 50	> 50
1c	27	13.52	> 50
1 d	29	> 50	> 50
1e	80	6.70	15.32
1 f	17	> 50	> 50
1 g	100	> 50	> 50
1 h	9	> 50	> 50
1i	98	> 50	> 50
1j	29	> 50	> 50
**Drugs**			
Rifampicin	**100**	**—**	—
Isoniazid	**100**	—	—
Miltefosina	—	**1.28 µg/mL**	**7.19 µg/mL**
Nifurtimox	—	**1.31 µg/mL**	**2.50 µg/mL**

#### 2.2.3 Structure–Activity Relationship (SAR)

Antimicrobial, anti‐mycobacterial, and anti‐protozoal data indicates that activity is strongly influenced by substituent effects on the core scaffold. Compounds bearing electron‐donating and/or moderately lipophilic groups (e.g., 1e and 1g) demonstrated enhanced broad‐spectrum antibacterial and anti‐mycobacterial activity, while derivatives such as 1c and 1e showed improved anti‐protozoal potency, suggesting that both electronic and lipophilic factors contribute to biological activity.

Compound 1g emerged as the most active anti‐mycobacterial and antibacterial agent, whereas 1e showed the best overall multi‐target profile, including antibacterial and anti‐protozoal activities. However, antifungal activity across the series remained generally weak.

Overall, the synthesized compounds exhibited moderate and selective pharmacological activity compared to standard drugs, with a few derivatives showing promising multi‐target potential. These findings identify compounds 1e and 1g as lead candidates, warranting further structural optimization to enhance potency and broaden their antimicrobial spectrum.

### 2.3 Molecular Docking

The integration of molecular docking in the present study was undertaken to provide a structural and mechanistic basis for the observed biological activity of the synthesized compounds. in silico approaches were employed to (i) predict the binding mode of the compounds, (ii) identify key interactions within the active site, and (iii) rationalize structure–activity relationships. The enzyme enoyl‐acyl carrier protein reductase (InhA) from Mycobacterium tuberculosis was selected as the molecular target due to its well‐established role in mycolic acid biosynthesis, a critical component of the mycobacterial cell wall. Inhibition of InhA disrupts cell wall formation, ultimately leading to bacterial death. Importantly, InhA is a validated target of first‐line antitubercular agents such as Isoniazid, further supporting its biological relevance. The crystal structure of InhA (PDB ID: 4TZK) was therefore utilized for docking studies [22, 23].

The docking scores of the synthesized compounds against *Mycobacterial* enoyl‐acyl carrier protein reductase (InhA; PDB ID: 4TZK) are presented in Table 3. All compounds exhibited favorable binding energies, with docking scores ranging from ‐7.124 to 8.929 kcal/mol. The co‐crystallized

**TABLE 3:** Docking score (in Kcal/mol) of the synthesized compound in active site of Mycobacterial enoyl reductase (InhA) (PDB ID: 4TZK).

Compound	Docking score
1a	−8.411
1b	−8.929
1c	−8.172
1 d	−7.132
**1e**	−8.458
1 f	−8.372
**1 g**	‐8.327
1 h	−7.124
1i	−8.258
1j	−7.725
Isoniazid	−6.458
Co‐crystal Ligand	−10.245

ligand showed the strongest affinity (−10.245 kcal/mol), serving as a benchmark for optimal binding. Among the synthesized series, compound 1b demonstrated the most favorable docking score (−8.929 kcal/mol), followed by 1e (−8.458 kcal/mol), 1a (−8.411 kcal/mol), and 1f (−8.372 kcal/mol). In comparison, the reference drug isoniazid exhibited a comparatively weaker docking score of −6.458 kcal/mol. Importantly, compound 1e, which showed complete growth inhibition at 50 µg/mL in the biological assay, also displayed a strong binding affinity toward InhA, indicating good concordance between in silico and in vitro findings. Likewise, compound 1 g exhibited a docking score of −8.327 kcal/mol and demonstrated 80% inhibition at the same concentration, further supporting its inhibitory potential.

**FIGURE 1:**
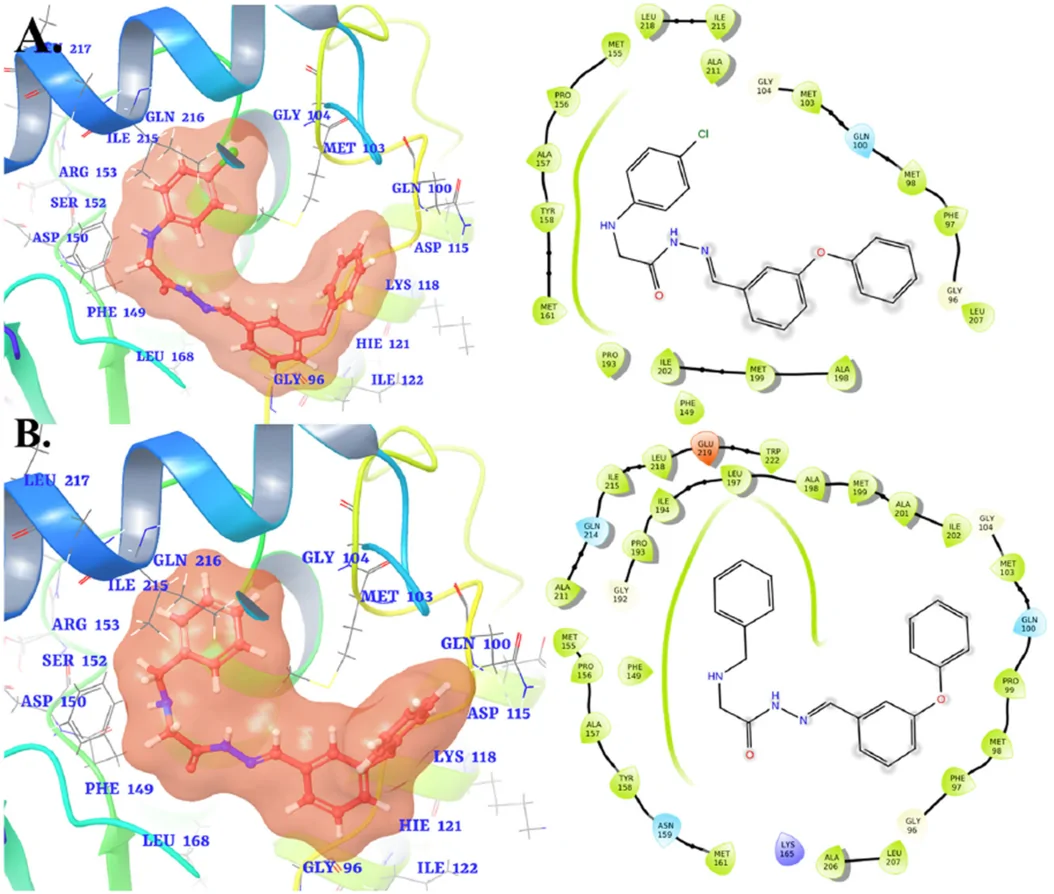
2D (left) and 3D (right) Biding mode of (A) compound **1e**, and (B) **1g** in the active site of *Mycobacterial enoyl reductase* (InhA) (PDB ID: 4TZK).

The predicted binding conformations of compounds 1e and 1 g within the InhA active site are illustrated in Figure 1. Interaction analysis revealed that ligand stabilization was predominantly mediated through van der Waals interactions involving key residues, including Gly96, Phe97, Met98, Gln100, Met103, Gly104, Pro156, Ala157, Tyr158, Met161, Met193, Leu197, Ala198, Met199, Ile202, Ala206, and Leu207. Although direct hydrogen bonding interactions were not observed, the collective contribution of these non‐covalent contacts facilitated favorable ligand orientation and stable accommodation within the binding pocket.

Overall, the docking results suggest that hydrophobic and van der Waals interactions play a crucial role in governing ligand binding and inhibitory activity against InhA. These findings substantiate the experimental observations and highlight the synthesized compounds, particularly 1e and 1g, as promising scaffolds for further optimization as antitubercular agents.

### 2.4 MD Simulation

Molecular dynamics (MD) simulations were conducted to investigate the conformational stability and dynamic behavior of compounds 1e and 1g in complex with *Mycobacterial* InhA. These simulations provided time‐dependent insights into ligand–protein interactions and structural adaptations within the binding site. The trajectories were analyzed using key parameters, including root mean square deviation (RMSD), root mean square fluctuation (RMSF), and ligand–protein contact profiles.

RMSD analysis was employed to assess the structural stability of the apo InhA protein and the ligand‐bound complexes over the 200 ns simulation period. RMSD values reflect the average atomic displacement relative to the initial conformation, with lower deviations indicative of greater structural stability [24, 25]. Comparative RMSD profiles revealed a similar trend for both ligand‐bound systems, characterized by an initial increase during the first 50 ns, followed by stabilization with minor fluctuations for the remainder of the simulation. This behavior indicates equilibration of the complexes and the attainment of stable binding conformations.

**FIGURE 2:**
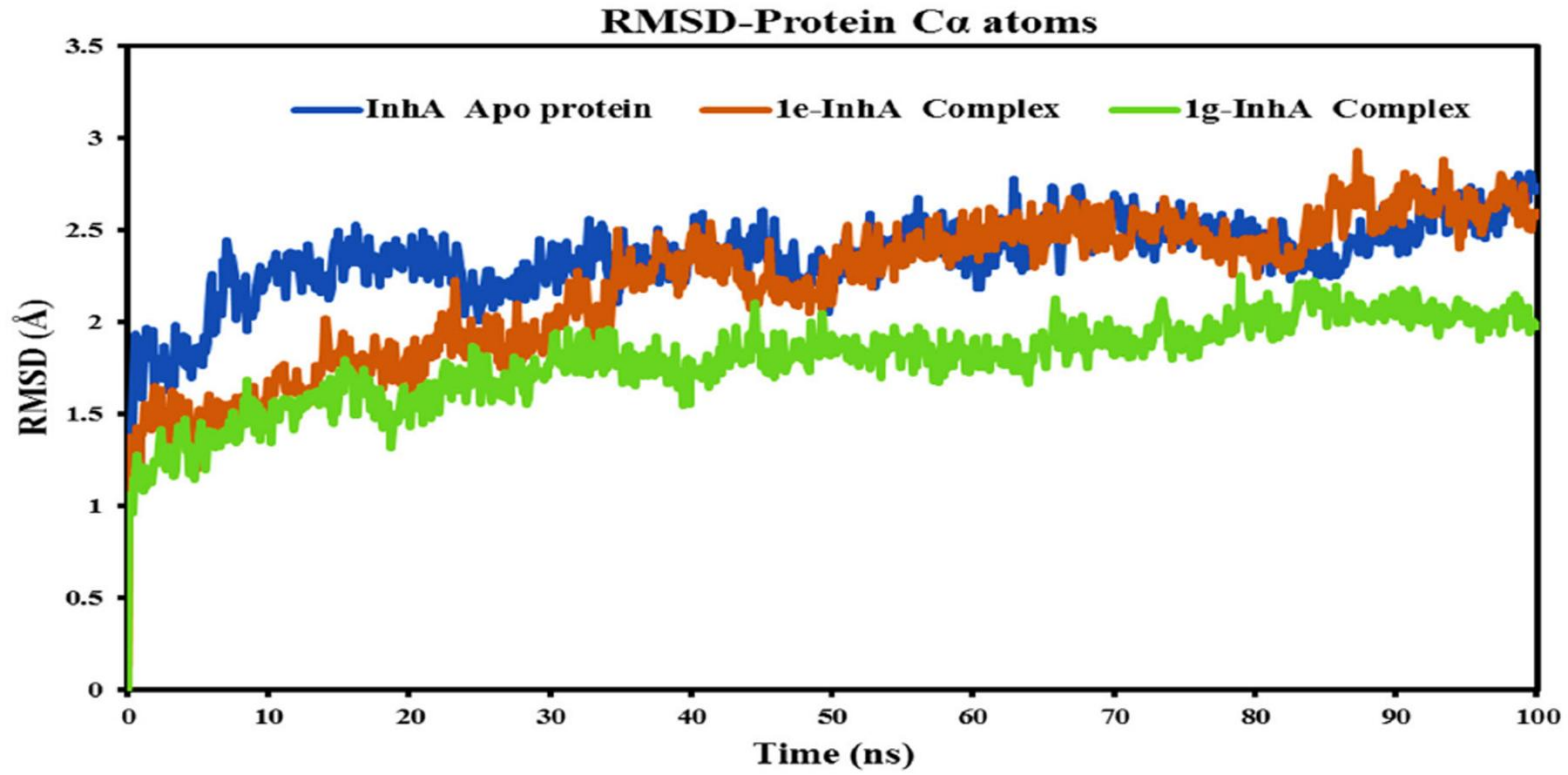
Time dependent RMSD of InhA Apo protein Cα atoms and in complex with compound **1e** and **1g**.

The 1e–InhA complex exhibited a minimum RMSD of 1.03 Å and an average RMSD of 2.20 Å, suggesting that compound 1e binds stably within the active site while inducing minimal perturbation to the overall protein structure. Similarly, the 1g–InhA complex demonstrated slightly lower RMSD values, with a minimum of 0.96 Å and an average of 1.79 Å, indicating enhanced conformational stability relative to the 1e–InhA complex. Despite minor fluctuations, the RMSD trajectory confirms persistent ligand engagement and structural integrity throughout the simulation Figure 2.

Collectively, the RMSD results indicate that both compounds form stable complexes with InhA, with compound 1g exhibiting marginally greater stability. These findings further support the docking results and reinforce the potential of compounds 1e and 1g as promising InhA inhibitors.

Throughout the 200 ns simulation, the RMSD values of both ligand‐bound systems remained within an acceptable range, confirming the overall stability of the protein–ligand complexes. These results indicate that compounds 1e and 1g are capable of forming stable associations with the InhA protein, requiring only minor conformational adjustments to accommodate ligand binding.

Root mean square fluctuation (RMSF) analysis was performed to assess residue‐level flexibility and dynamic behavior of InhA in its apo form and in complex with compounds 1e and 1g. RMSF provides insight into localized structural mobility by monitoring fluctuations of individual amino acid residues over the simulation trajectory [26, 27]. For the apo InhA protein, RMSF values ranged from 0.46 to 4.65 Å, with an average of 1.07 Å, indicating overall structural stability accompanied by increased flexibility in specific regions. These flexible regions are likely associated with functional elements of the protein, such as active‐site loops or regions involved in molecular recognition.

In the 1e–InhA complex, a marginal increase in residue flexibility was observed, with RMSF values ranging from 0.41 to 5.10 Å and an average of 1.15 Å. This slight elevation suggests that binding of compound 1e induces localized conformational adaptability without compromising the global stability of the protein structure.

The reduction in the minimum RMSF value observed for the 1e–InhA complex indicates that certain residues exhibit decreased fluctuations as a result of stabilizing interactions with the bound ligand. In contrast, the 1g–InhA complex demonstrates a more pronounced stabilizing effect on the protein. RMSF analysis of this complex revealed values ranging from 0.38 to 4.16 Å, with an average of 0.99 Å, indicating a reduction in overall residue flexibility relative to both the apo protein and the 1e–InhA complex. These results suggest that compound 1g confers enhanced conformational rigidity to InhA upon binding.

**FIGURE 3:**
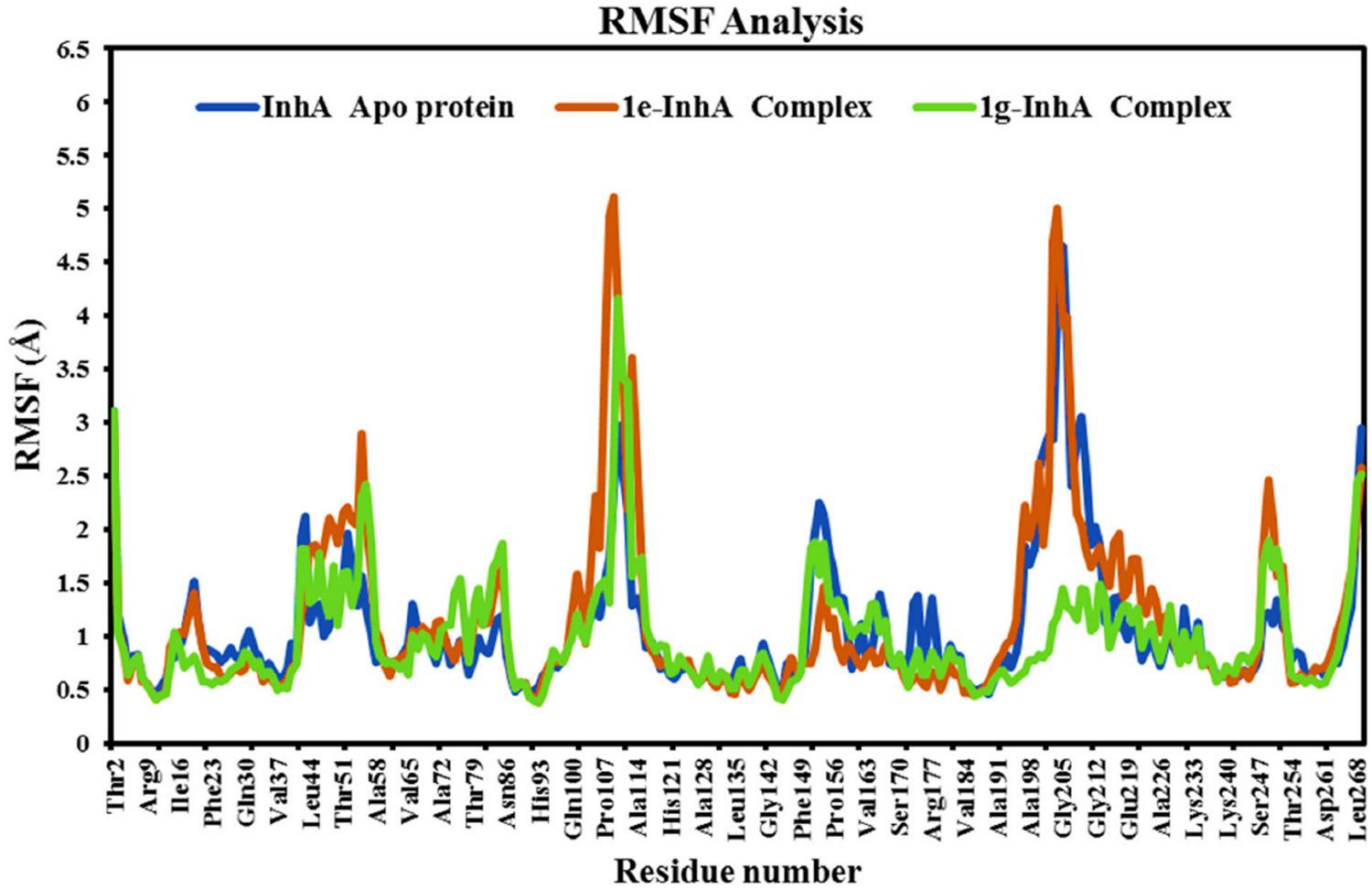
RMSF of individual amino acids of InhA Apo protein Cα atoms and in complex with compound **1e** and **1g**.

During the simulation, compound 1g maintained persistent interactions with a defined set of amino acid residues, including Gly14, Ile15, Ile16, Thr17, Ser19, Ser20, Ile21, Ala22, Gly40, Phe41, Asp42, Arg43, Leu63, Asp64, Val65, Gln66, His93, Ser94, Ile95, Gly96, Phe97, Met98, Pro99, Gln100, Met103, Gly104, Ile105, Lys118, Ile122, Ser123, Met147, Asp148, Phe149, Met155, Ala157, Tyr158, Met161, Lys165, Ala191, Gly192, Pro193, Ile194, Arg195, Thr196, Leu197, Ala198, Met199, Ala201, Ile202, Ala206, Leu207, Ile215, Leu218, and Met232 (Figure 3).

Notably, the RMSF values of these interacting residues were comparatively lower, underscoring their contribution to stabilizing the ligand–protein interface. This coordinated reduction in residue‐level flexibility highlights the structural robustness of the 1g–InhA complex and further supports the superior binding stability of compound 1g observed during the MD simulation.

Hydrogen bond analysis during MD simulations provides critical insights into the binding interactions between ligands and their target protein. Assessment of the number, occupancy, and stability of hydrogen bonds enables evaluation of interaction strength, specificity, and potential efficacy of the complex. Persistent hydrogen bonding with high occupancy throughout the simulation indicates stable ligand–protein interactions and contributes to maintenance of the ligand within the binding site [28, 29, 30, 31, 32]. Although hydrogen bonding was not observed in the initial docking poses, MD simulations revealed the formation of transient hydrogen bonds over time, likely due to protein flexibility and solvent‐mediated interactions, indicating that such interactions are dynamic and may not be captured in rigid docking analysis.

**FIGURE 4:**
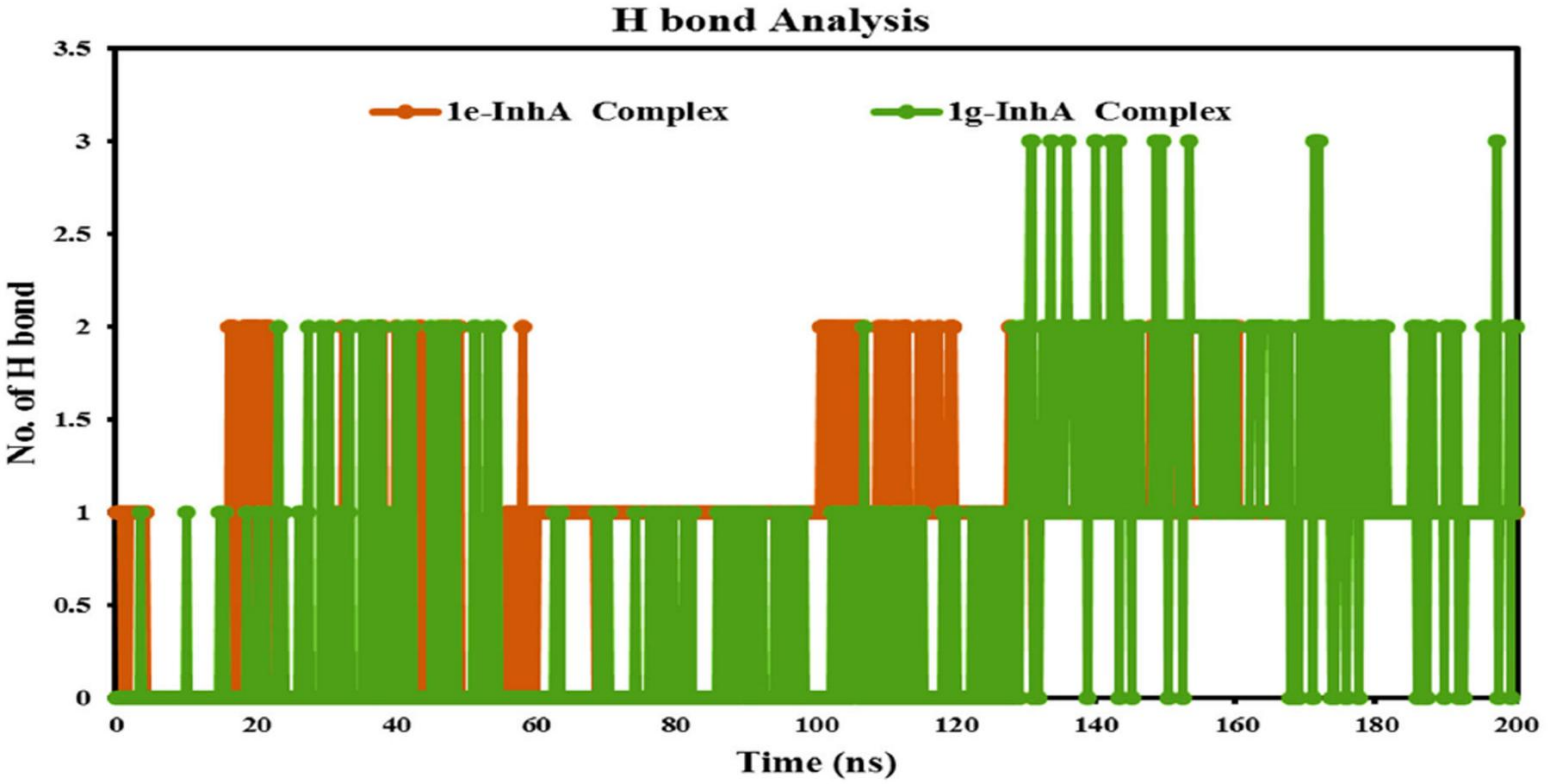
Time dependent hydrogen bond analysis of compound **1e** and **1g** in complex with InhA protein.

The hydrogen bond profiles of the 1e–InhA and 1g–InhA complexes are shown in Figure 4. For the 1e–InhA complex, the number of hydrogen bonds ranged from 1 to 2, with an average of 0.83. This suggests that at least one hydrogen bond was consistently maintained throughout the simulation, with intermittent formation of a second bond, indicating a notable role of hydrogen bonding in stabilizing the complex.

In contrast, the 1g–InhA complex exhibited 1–3 hydrogen bonds during the simulation, with an average of 0.63. Although this complex occasionally formed up to three hydrogen bonds, these interactions were less consistent than in the 1e–InhA system. These findings suggest that while hydrogen bonding contributes to stability in both complexes, the 1e–InhA complex demonstrates relatively more persistent hydrogen‐mediated interactions.

## 3 Materials and Methods

Melting points of all synthesized compounds were determined using the open capillary method and are uncorrected. Reaction progress was monitored by thin‐layer chromatography (TLC) on silica gel, visualized under UV light or with iodine vapor. Infrared (IR) spectra were recorded on Agilent Resolution Pro FT‐IR and Perkin Elmer RZX spectrophotometers using KBr pellets, with frequencies reported in cm^−1^. ^1H and ^13 C NMR spectra were acquired on a Bruker Advance II 400 MHz spectrometer using DMSO‐d_6_ or CDCl_3_ as solvent and tetramethylsilane (TMS) as internal standard. Mass spectra were recorded on a WATERS Q‐TOF micro mass spectrometer (ESI‐MS). Elemental analyses (C, H, N) were performed on a Carlo Erba 1180 CHN analyzer.

*General Synthesis procedure of A (1‐10): ethyl‐2‐(substituted‐arylamino)acetate*

An aromatic amine (0.1 mol) was dissolved in dry DMF (25 mL), and anhydrous potassium carbonate (2.5 mol) and ethyl chloroacetate (1.25 mol) were added. The mixture was refluxed at 80°C for 15–17 h. Reaction progress was monitored by TLC using ethyl acetate:toluene (3:7 v/v). After cooling, the mixture was poured into water, and the precipitated solid was collected, washed, dried, and recrystallized from methanol to afford the purified esters.

*Synthesis of B (11‐20): 2‐(substituted‐arylamino) acetohydrazide*

Ethyl‐2‐(substituted‐arylamino)acetate (0.05 mol) in methanol (30 mL) was treated with hydrazine hydrate (0.1 mol) and refluxed for 10–12 h. TLC (ethyl acetate:toluene, 2:8 v/v) monitored the reaction. After solvent removal under reduced pressure, the crude product was recrystallized from acetone and washed with water to yield the acetohydrazides.

*Synthesis of (Z)‐2‐(substituted‐arylamino)‐N’‐(3‐phenoxybenzylidene) acetohydrazide: 1(a‐j)*

2‐(Substituted‐arylamino)acetohydrazide (1.0 mol) in methanol (30 mL) was treated with m‐phenoxybenzaldehyde (1.5 mol) and a few drops of glacial acetic acid. The mixture was refluxed for 6–8 h. Reaction progress was monitored by TLC (methanol:dichloromethane, 1:9 v/v). After solvent removal under reduced pressure, the crude product was poured into cold water, filtered, dried, and recrystallized from suitable alcohol to afford the target hydrazides.

**2‐(4, 6‐dimethoxypyrimidin‐2‐ylamino)‐*N*’‐(3‐phenoxybenzylidene)aceto‐hydrazide (1a):**

**Yield: 60%, M.P.: 252 C°, IR** (cm^−1^): 3255 (‐NH stretching), 2929.14 (CH‐; asym, sym), 1680.17 (C = O, amide), 1587.18 (C = N‐Schiff's base), 1242.02 (aryl ether). ^**1**^ **HNM**R (δppm): 8.5 (s, 1H, CH = N‐), 8.40 (s, 1H, CONH)), 7.1‐7.9 (m, 9H, Ar‐H), 5.82 (s, 1H, ‐CH‐pyrimidine), 4.34 (s, 1H, ‐ NH‐CH_2_), 3.96 (s, 6H, ‐OCH_3_‐pyrimidine), 3.82 (s, 2H, CO‐CH_2_‐); ^**13**^ **CNMR**: 171.9 (CONH), 169.8 (C_4_, C_6_‐pyrimidine), 159‐158 (C‐O‐C, diaryl ether), 145.9 (Ar‐C = N‐), 117.8‐137.6 (Ar‐C), 95.8 (C_5_‐pyrimidine), 57.9 (CO‐CH_2_), 55.8 (‐OCH_3_‐pyrimidine); **MS (EI)** *m*/*z*: 391 (M^+^), 392 (M + 1).

**N’‐(3‐phenoxybenzylidene)‐2‐(piperazin‐1‐ylamino)acetohydrazide (1b):**

**Yield: 55%, M.P.: 230 C°,** IR (cm^−1^): 3267 (‐NH stretching), 2919.17 (CH‐; asym, sym), 1645.10(C = O, amide), 1598.28 (C = N‐Schiff's base), 1212.09 (aryl ether). ^**1**^ **HNMR** (δppm): 8.69 (s, 1H, (hydrazide NH), 8.42 (s, 1H, CH = N), 8.11 (s, 1H, CONH), 7.1‐7.6 (m, 9H, Ar‐H), 3.67 (m, 4H, piperazine N–CH_2_), 3.18 (s, 2H, –CO–CH_2_–), 2.58 (m, 4H, piperazine CH_2_), 1.77 (s, 1H, Piperazine –NH, disappear in D_2_O) ^**13**^ **CNMR:** 171.93 (C = O), 157.26, 156.11 (C = N (azomethine) & Ar‐O‐C), 150‐158 (C‐O‐C, diarylether), 116.7‐143.9 (Ar‐C), 59.9 (CO‐CH_2_), 52–45 (Piperazine Carbon); MS (EI) m/z: 353 (M + ), 354 (M + 1).

**N’‐(3‐phenoxybenzylidene)‐2‐(p‐tolylamino)acetohydrazide (1c):**

**Yield: 65%, M.P.: 202 C°, IR** (cm^−1^): 3289 (‐NH), 3029.10 (CH‐; asym, sym), 1655.87 (C = O, amide), 1597.13 (C = N‐Schiff's base), 1540.78 (Aromatic C = C), 1245.09 (aryl ether). ^**1**^ **HNM**R (δppm): 8.35 (s, 1H, CH = N‐), 7.6‐7.4 (m, 13H, Ar‐H), 5.20 (s, 1H, ‐NH‐CO), 4.15 (s, 2H, –CO–CH_2_), 3.20 (s, 1H, –NH–Ar), 2.30 (s, 3H, Ar–CH_3_, p‐tolyl) 3.82 (s, 2H, CO‐CH_2_‐); ^**13**^ **CNMR**: 171.9 (CONH), 158.09‐159.8 (C‐O‐C, diarylether), 145.9 (Ar‐C = N‐), 117.8‐137.6 (Ar‐C), 57.9 (CO‐CH_2_), 20.1 ppm (Ar–CH_3_) **MS (EI)** *m*/*z*: 359 (M^+^), 360 (M + 1).

**2‐(1,5‐dimethyl‐3‐oxo‐2‐phenyl‐2,3‐dihydro‐1** ***H*** **‐pyrazol‐4‐ylamino)‐** ***N’*** **‐(3‐phenoxy benzylidene) acetohydrazide (1d):**

**Yield: 65%, M.P.: 246 C°, IR** (cm^−1^): 3175 (‐NH), 2929.23 (CH‐; asym, sym), 1688.17(C = O, amide), 1665.32(C = O, pyrazolone), 1581.08(C = N‐Schiff's base), 1242.22 (aryl ether). ^**1**^ **HNMR** (δppm): 8.65 (s, 1H, CH = N‐), 8.32 (s, 1H, CONH), 6.98‐7.75 (m, 14H, Ar‐H), 3.98 (s, 2H, CO‐CH_2_), 3.75 (s, 3H,‐CH_3_‐N‐pyrazolone), 2.87 (s, 3H, ‐CH_3_‐pyrazolone), 2.1 (s, 1H, ‐NH‐pyrazolone); ^**13**^ **CNMR**: 168.9 (CONH), 165.8 (C = O‐pyrazolone), 159‐160 (C‐O‐C, diarylether), 143.9 (Ar‐C = N), 136.4 (C_5_‐Pyrazolone), 117.8‐137.6(Ar‐C), 115.8(C_4_‐pyrazolone), 55.9(CO‐CH_2_), 32.8(‐CH_3_‐N‐pyrazolone), 26.8(‐CH_3_‐pyrazolone); **MS** (EI) *m*/*z*: 455 (M^+^), 456 (M + 1).

**2‐((4‐chlorophenyl)amino)‐N’‐(3‐phenoxybenzylidene)acetohydrazide (1e):**

**Yield: 62%, M.P.: 215 C°, IR** (cm^−1^): 3288.12 (‐NH), 2929.30 (CH‐; asym, sym), 1654.76 (C = O, amide), 1585.34 (C = N‐Schiff's base), 1540.86 (Aromatic C = C), 1248.90 (aryl ether), 755.67 C–Cl stretching (para‐substituted ring). **1HNMR** (δppm): 11.4 (s, 1H, (hydrazide NH), 8.45 (s, 1H, CH = N), 7.4‐7.1 (m, 13H, Ar‐H), 4.20 (s, 1H, ‐NH‐CO), 3.20 (s, 1H, –NH–Ar), 3.8 (s, 2H, CO‐CH_2_‐); ^**13**^ **CNMR**: 172.6 (CONH), 157‐159 (C‐O‐C, diarylether), 145.7(Ar‐C = N‐), 116.7‐137.8 (Ar‐C), 57.8 (CO‐CH_2_), **MS (EI)** *m*/*z*: 379.11 (M^+^), 380.11 (M + 1) 381.11 (M + 2).

**2‐((3‐chloro‐5‐methylphenyl)amino)‐N’‐(3‐phenoxybenzylidene)acetohydrazide (1f):**

**Yield: 68%, M.P.: 223 C°, IR** (cm^−1^): 3300.12 (‐NH), 2920.33 (CH‐; asym, sym), 1667.70 (C = O, amide), 1600.34 (C = N‐Schiff's base), 1560.56 (Aromatic C = C), 1240.90 (aryl ether), 750.77 C–Cl stretching (substituted aromatic ring). **1HNMR** (δppm): 11.3 (s, 1H, (hydrazide NH), 8.20 (s, 1H, CH = N‐), 7.9‐6.4 (m, 13H, Ar‐H), 3.47 (s, 1H, ‐NH‐CO), 3.20 (s, 1H, –NH–Ar), 2.35 (s, 3H, Ar–CH_3_, 3‐chloro‐5‐methylphenyl) 3.8 (s, 2H, CO‐CH_2_‐); ^**13**^ **CNMR**: 170.6 (CONH), 150‐160 (‐Ar‐C = N‐& aromatic C‐O), 115.7‐130.8 (Ar‐C), 55.5‐60.3 (CO‐CH_2_), 25.34 (‐CH_3_) **MS (EI)** *m*/*z*: 394 (M^+^), 395 (M + 1) 396 (M + 2).

**2‐(benzylamino)‐N’‐(3‐phenoxybenzylidene)acetohydrazide (1g):**

**Yield: 60%, M.P.: 205 C°, IR** (cm^−1^): 3380 (‐NH), 2929.16 (CH‐; asym, sym), 1650.80 (C = O, amide), 1590.12 (C = N‐Schiff's base), 1549.00 (Aromatic C = C), 1244.03 (aryl ether). ^**1**^ **HNM**R (δppm): 11.1 (s, 1H, ‐NH‐CO), 8.4 (s, 1H, CH = N‐), 7.4‐7.1 (m, 13H, Ar‐H), 6.20 (s, 1H, –NH–Ar), 3.54 (s, 2H, CO‐CH_2_‐), 4.25 ppm (2H, s or slightly broadened); ^**13**^ **CNMR**: 171.9‐165.3 (CONH), 155‐150 (C‐O‐C, diarylether), 155.9 (Ar‐C = N‐, overlaps), 117.8‐130.6(Ar‐C), 50.9 (CO‐CH_2_), 42 (‐benzyl CH_2_‐) **MS (EI)** *m*/*z*: 360 (M^+^) 361.1 (M + 1).

***N’*** **‐(3‐phenoxybenzylidene)‐2‐(** ***1H*** **‐1,2,3‐triazol‐1‐yl)acetohydrazide (1h):**

**Yield: 68%, M.P.: 195 C° IR** (cm^−1^): 3252 (‐NH), 2928.14 (CH‐; asym, sym), 1666.17 (C = O, amide), 1577.19 (C = N‐Schiff's base), 1399.10 (1,2,3‐triazole),1217.20 (aryl ether). ^**1**^ **HNMR** (δppm): 8.55(s, 1H, CH = N‐), 8. 20 (s, 1H, CONH), 7.7‐8.1 (s, 1H, triazole‐H), 7.75‐7.12(m, 9H, Ar‐H), 5.98 (s, 2H, CO‐CH_2_); ^**13**^ **CNMR**: 166.5 (CONH), 159‐160 (C‐O‐C, diarylether), 143.9 (Ar‐C = N‐), 136.4 (C_4_‐triazole), 126.2 (C_5_‐triazole), 117.8‐137.6 (Ar‐C), 63.9 (CO‐CH_2_); **MS** (EI) *m*/*z*: 321.5 (M^+^).

**2‐(2‐(5‐methylbenzo[d]thiazol‐2‐yl)hydrazinyl)‐N’‐(3‐phenoxybenzylidene)acetohydrazide(1i):**

**Yield: 67%, M.P.: 209 C° IR** (cm^−1^): 3255 (‐NH), 3046.13 (CH‐; asym, sym), 1620.67 (C = O, amide), 1590.78 (C = N‐Schiff's base), 1530.48 (Aromatic C = C), 1240.20 (aryl ether), 710.34 (C‐S, benzothiazole). ^**1**^ **HNMR** (δppm):10.85 (s, 1H, hydrazide) 8.45 (s, 1H, CH = N‐), 8.30 (s, 1H, CONH), 7.42‐7.05 (m, 9H, Ar‐H), 4.34 (br s, 1H, –NH–, exchangeable), 3.54 (s, 2H, COCH_2_), 2.47 (s, 3H, ‐CH_3_) ^**13**^ **CNMR**:170.5 (C = O), 150–165 (C = N and heteroaromatic carbons), 115–140 (Ar–C), 50.9 (–CO–CH_2_–), 22.8 (–CH_3_);**MS** (EI) *m*/*z*: 401.11(M^+^) 402 (M + 1).

**2‐(5‐methylthiazol‐2‐ylamino)‐** ***N’*** **‐(3‐phenoxybenzylidene) acetohydrazide (1j):**

**Yield: 70%, M.P.: 210 C° IR** (cm^−1^): 3254 (‐NH stretching), 2929.14 (CH‐; asym, sym), 1665.17 (C = O, amide), 1575.19 (C = N, Schiff's base), 1499.10 (1,2,3‐thizole),1227.20 (aryl ether). ^**1**^ **HNMR** (δppm): 8.35(s, 1H, CH = N‐), 8.0 (s, 1H, CONH)), 7.18‐7.75 (m, 9H, Ar‐H), 6.78 (s, 1H,‐CH‐thiazole), 4.34 (br s, 1H, NH‐Ar), 3.87 (s, 2H, COCH_2_), 2.87 (s, 3H, ‐CH_3_‐thiazole) ^**13**^ **CNMR**: 174.5 (CONH), 163.5 (C_2_‐thiazole), 159‐160 (C‐O‐C, diarylether), 143.9 (Ar‐C = N‐), 135.4 (C_4_‐thiazole), 123.2 (C_5_‐thiazole), 117.8‐137.6 (Ar‐C), 57.9 (CO‐CH_2_), 27.8 (‐CH_3_‐thiazole); **MS** (EI) *m*/*z*: 366 (M^+^), 367 (M + 1).

### 3.1 Pharmacology

The antimicrobial potential of compounds 1a–1j was evaluated quantitatively using the minimum inhibitory concentration (MIC) method against a panel of bacterial and fungal strains. MIC determination was performed using the broth microdilution technique, in which the test organism is exposed to serial dilutions of the compound in a nutrient broth [33, 34]. The MIC is defined as the lowest concentration of the compound that completely inhibits visible microbial growth under the experimental conditions. Microdilution assays were conducted in a microtiter plate format, with a total broth volume of 0.05–0.1 mL per well.

The antibacterial and antifungal activities were assessed against two Gram‐positive bacteria—*Staphylococcus aureus* (MTCC‐96) and *Streptococcus pyogenes* (MTCC‐443); two Gram‐negative bacteria—*Escherichia coli* (MTCC‐442) and *Pseudomonas aeruginosa* (MTCC‐441); and three fungal strains—*Candida albicans* (MTCC‐227), *Aspergillus niger* (MTCC‐282), and *Aspergillus clavatus* (MTCC‐1323). All microbial strains were obtained from the Institute of Microbial Technology (Chandigarh, India).

#### 3.1.1 In Vitro Anti‐Mycobacterial Activity

L. J. Slope employed the broth microdilution method, following the guidelines of the National Committee for Clinical Laboratory Standards, to determine the minimum inhibitory concentrations (MICs) in an anti‐mycobacterial study. Dimethyl sulfoxide (DMSO) was used as both the solvent and vehicle to achieve the desired compound concentrations for testing against standard bacterial strains [35].

#### 3.1.2 In Vitro Anti‐Protozoa Activity

The growth inhibitory effects of the synthesized compounds were evaluated against *Trypanosoma cruzi* epimastigotes (MHOM/MX/1994/NINOA; clinical strain from an acute‐phase patient) and *Leishmania mexicana* promastigotes (MHOM/MX/ISETGS; clinical strain from a patient with diffuse cutaneous leishmaniasis). Parasites were cultured at 26°C in Schneider's Drosophila medium supplemented with 100 IU/mL penicillin, 100 µg/mL streptomycin, and 10% fetal bovine serum.

Compounds were tested in duplicate using 96‐well microplates. Stock solutions were prepared in DMSO and diluted in culture medium. Each well received 100 µL of compound solution and 100 µL of parasite suspension containing 10,000 *Leishmania* promastigotes or 20,000 *T. cruzi* epimastigotes, yielding final compound concentrations of 50, 25, 12.5, 6.25, and 3.125 µg/mL. Benznidazole and miltefosine were included as positive controls, and wells without compounds served as negative controls. Following a 72 h incubation at 26°C, parasite counts were performed using a Neubauer chamber. The concentration required to inhibit 50% of parasite growth (IC_50_) was determined from the resulting dose–response curves [36].

### 3.2 Methodology

#### 3.2.1 Molecular Docking and Dynamic (MD) Simulation Studies

Molecular docking of the newly synthesized compounds was performed against *Mycobacterial* enoyl reductase (InhA), using the crystal structure with PDB ID 4TZK. Ligands were prepared using the LigPrep tool, generating possible enantiomers, ionization states, and tautomeric forms at pH 7.0 ± 2, followed by energy minimization employing the OPLS3e force field [37, 38].

Protein preparation was carried out using the Protein Preparation Wizard, which included removal of crystallographic water molecules, assignment of bond orders and partial charges, determination of residue ionization and tautomeric states, and optimization of hydrogen‐bonding networks. The receptor grid was defined using the co‐crystallized Pyrrolidine carboxamide inhibitor, resulting in a default grid box with dimensions X = 8.93, Y = 32.47, and Z = 60.42 Å. Docking simulations were performed in the active site using the standard precision (SP) Glide protocol, with all docking and scoring parameters maintained at default settings [39, 40].

#### 3.2.2 MD Simulation

Atomistic molecular dynamics (MD) simulations were performed to investigate the dynamic interactions between the ligands and InhA over time. Each protein–ligand complex was solvated in a periodic orthorhombic box with a 10 Å buffer between the solute and the box boundaries. The system was modeled using the single‐point charge (SPC) water model, with Na^+^ and Cl^−^ counterions added to neutralize the system and achieve an ionic strength of 0.15 M NaCl, mimicking physiological conditions. System setup was carried out using the Desmond System Builder panel.

The solvated complexes were subjected to energy minimization and relaxation according to the default Desmond protocol with the OPLS3e force field, ensuring removal of steric clashes and proper system equilibration. Production MD simulations were then performed for 200 ns under the NPT ensemble at 300 K and 1.013 bar, with default relaxation settings applied prior to data collection. Trajectory analyses, including root mean square deviation (RMSD), root mean square fluctuation (RMSF), and protein–ligand interactions, were performed using the Simulation Interaction Diagram module in Maestro [41, 42, 43, 44, 45, 46].

All simulations were executed on a Linux‐based system (Ubuntu 22.04.2 LTS, 64‐bit) equipped with an Intel Xeon W‐2245 CPU (3.90 GHz, 8 cores) and an NVIDIA RTX A4000 GPU with CUDA 12, providing the necessary computational performance for extended MD runs.

## 4 Conclusions

**FIGURE 5:**
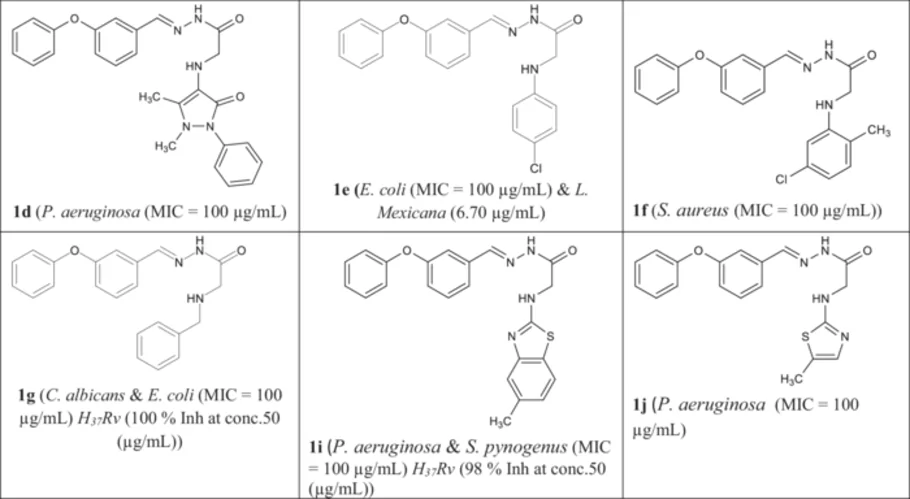
Structure of active molecules of this series.

The antibacterial, antitubercular, and antiprotozoal properties of a number of Schiff base analogues based on biphenyl ether were successfully synthesized and assessed in this work. **Compounds 1g** and **1i** had significant anti‐TB efficacy against Mycobacterium tuberculosis H37Rv, with substantial inhibition at 50 µg/mL. Compounds **1e** and **1c** showed moderate antiprotozoal efficacy, especially against Trypanosoma cruzi and Leishmania mexicana. The experimental results were corroborated by molecular docking investigations, which revealed that a number of compounds (most notably **1a**, **1b**, and **1g**) had favorable binding affinities when compared to the common medication isoniazid, indicating a potential relationship between binding interactions and biological activity. Substituents like methoxy, chloro, methyl groups, and heterocyclic moieties (like Piperazine, pyrazole, thizole, benzothizole) may affect biological activity, according to preliminary structure–activity relationship study. These results indicate that Schiff base compounds based on biphenyl ether offer a viable scaffold for future development, especially for antitubercular and antiprotozoal uses, necessitating more thorough biological and mechanistic studies. The chemical structures of the active compounds are depicted below in Figure 5.

## Author Contributions

**Navin B. Patel:** resources, funding acquisition. **Deepti S. Ghaisas:** investigation, methodology. **Vatsal M. Patel:** formal analysis. **Faiyazalam M. Shaiikh:** formal analysis. **Mohit Agrawal:** resources. **Mohd Imran:** funding acquisition. **Gildardo Rivera:** formal analysis. **Hetal I Soni:** writing – original draft, writing – review and editing.

## Conflicts of Interest

The authors declare no conflicts of interest.

## Supporting information


Supporting File

## Data Availability

The data supporting the findings of this study are included within the article and its supplementary material.
