# Supplementary material for: Biological Evaluation, Molecular Docking, MD Simulation Study and Synthesis of (Z)‐N’‐(3‐phenoxybenzylidene)‐2‐(substituted‐amino aryl) Acetohydrazid
Source: J Biochem Mol Toxicol. 2026 Sep 23;40(10):e71114. doi: 10.1002/jbt.71114 (PMC13600652; doi:10.1002/jbt.71114)
Supplement: Supplementary file 1 [file JBT-40-e71114-s001.docx]

**“Biological evaluation, Molecular docking, MD simulation study and Synthesis of (*Z)-N'*-(3-phenoxybenzylidene)-2-(substituted-amino aryl) acetohydrazid.”**

**Navin B. Patel^1^, Deepti S. Ghaisas^1^, Hetal I. Soni^2*^, Mohit Agrawal^3^, Siddique Akber Ansari^4^, Gildardo** **Rivera^5^, Hiral Tailor^6^**

**Supporting information**

| **Figure** | **List of figures** | **Page number** |
| --- | --- | --- |
| 1 | IR spectrum of intermediate 2 | 2 |
| 2 | IR spectrum of intermediate 8 | 2 |
| 3 | I IR spectrum of intermediate 15 | 3 |
| 4 | ^1^H NMR of intermediate 15 | 3 |
| 5 | ^13^C NMR of intermediate 15 | 4 |
| 6 | ^1^HNMR of **1b** | 4 |
| 7 | IR spectrum of **1d** | 5 |
| 8 | Mass Spectrum of **1h** | 5 |
| 9 | ^13^C NMR of **1b** | 6 |

**IR of intermediate 2**

**IR of intermediate 8**

**IR of intermediate 15**

**^1^HNMR of intermediate 15**

**^13^CNMR of intermediate 15**

**^1^HNMR of comp1b**

**IR of comp 1d**

**MS of comp 1h**

**MS (EI) *m*/*z*:** 321 (M^+^), 322 (M + 1)

**^13^C NMR of 1b**
